# bioNerDS: exploring bioinformatics’ database and software use through literature mining

**DOI:** 10.1186/1471-2105-14-194

**Published:** 2013-06-15

**Authors:** Geraint Duck, Goran Nenadic, Andy Brass, David L Robertson, Robert Stevens

**Affiliations:** 1 School of Computer Science, The University of Manchester, Manchester, UK; 2 Computational and Evolutionary Biology, Faculty of Life Sciences, The University of Manchester, Manchester, UK; 3 Manchester Institute of Biotechnology, Manchester, UK

## Abstract

**Background:**

Biology-focused databases and software define bioinformatics and their use is central to computational biology. In such a complex and dynamic field, it is of interest to understand what resources are available, which are used, how much they are used, and for what they are used. While scholarly literature surveys can provide some insights, large-scale computer-based approaches to identify mentions of bioinformatics databases and software from primary literature would automate systematic cataloguing, facilitate the monitoring of usage, and provide the foundations for the recovery of computational methods for analysing biological data, with the long-term aim of identifying best/common practice in different areas of biology.

**Results:**

We have developed bioNerDS, a named entity recogniser for the recovery of bioinformatics databases and software from primary literature. We identify such entities with an F-measure ranging from 63% to 91% at the mention level and 63-78% at the document level, depending on corpus. Not attaining a higher F-measure is mostly due to high ambiguity in resource naming, which is compounded by the on-going introduction of new resources. To demonstrate the software, we applied bioNerDS to full-text articles from BMC Bioinformatics and Genome Biology. General mention patterns reflect the remit of these journals, highlighting BMC Bioinformatics’s emphasis on new tools and Genome Biology’s greater emphasis on data analysis. The data also illustrates some shifts in resource usage: for example, the past decade has seen R and the Gene Ontology join BLAST and GenBank as the main components in bioinformatics processing.

**Abstract:**

Conclusions We demonstrate the feasibility of automatically identifying resource names on a large-scale from the scientific literature and show that the generated data can be used for exploration of bioinformatics database and software usage. For example, our results help to investigate the rate of change in resource usage and corroborate the suspicion that a vast majority of resources are created, but rarely (if ever) used thereafter. bioNerDS is available at http://bionerds.sourceforge.net/.

## Background

The fields of bioinformatics and computational biology are established as ones of rapid change with a continued expansion of the available “resourceome” [1], which includes numerous databases and software [1,2]. Such resources facilitate research in biology, and many have become “household names” (e.g., BLAST [3], ClustalW [4], etc.). Still, the huge resourceome also creates problems for the choice of appropriate methods for performing a particular task, and poses a challenge of identifying “best practice”: a well-known, popular tool may not be the “best” tool currently available [5]. To help with method choice, we first need to determine what software and data resources are available and used in computational analyses. Several inventories and repositories already exist that list available database and software resources. For example, the 2011 special issues of *Nucleic Acids Research*’s Databases [6] and the Bioinformatics Links Directory [7] list over 1,330 databases and over 1,250 web services respectively. However, many of these inventories and repositories are incomplete and require labour intensive manual curation. Similarly, “manual” literature surveys of published tools and databases are time-consuming and often out-of-date by the time they are published. Therefore, large-scale automated ways for extraction of database and software use patterns are needed. As well as helping with maintenance of resource catalogues, such systematic processing could offer insights into the dynamics of software and data resource usage, particularly as many resources are infrequently used [2]. This is not only of interest to users of these resources, who wish to know what is current and most used, but also to any potential new users and resource developers.

In our previous work we used the literature to explore and evaluate methods used in phylogenetics [5,8]. We implemented a named-entity recognition (NER) system that utilised a controlled vocabulary of terms as specified by a comprehensive software resource dictionary. We also used a semantic-based approach to identify and profile existing and new resources using keyword association [8]. We then attempted to capture phylogenetic methods based on a predefined abstract representation of four stages within phylogenetics (sequence alignment; tree inference; statistical testing and data re-sampling; tree visualisation and annotation [5]). This approach could be applicable to other fields within bioinformatics, but it first requires an extensive resource repository and the ability to identify mentions of tools and databases in text. This task is far from trivial — in our previous work we have demonstrated the high level of ambiguity and variability of database and software names in the bioinformatics literature [9].

In this paper we introduce and evaluate bioNerDS, a bioinformatics named-entity recognition system for database and software names, which is used to identify mentions of such entities in the literature. It makes use of a range of both sentence and document-level clues to learn database and software names, while propagating mentions up to the article level. To illustrate its potential, we use bioNerDS to survey software and data resource usage in two journals from computational biology and bioinformatics.

Several other approaches to automated extraction of bioinformatics resources from primary literature have been suggested. For example, OReFiL (Online Resource Finder in Life sciences) [10] and BIRI (BioInformatics Resource Inventory) [11] aim to harvest resource names and fill their repositories in order to enable resource discovery. OReFiL uses URLs as a “proxy” to identify mentions of resources by custom regular expressions and by extracting |<url>.. </url>| tags from BioMed Central (BMC) papers. BIRI utilises keywords and sentence structure to identify relevant terms through custom patterns translated into “transition networks”, which match associated regular expressions for resource names, functions and classifications. bioNerDS on the other hand builds on established approaches to NER by using a generally applicable method for identification of software and database mentions. Furthermore, while OReFiL focuses on the abstract and “availability” or “implementation” sections, and BIRI solely looks into abstracts and titles, bioNerDS can detect resource name mentions throughout full-text articles.

We note that throughout this paper we will mention numerous databases and tools by name as examples. A full list of references and web-links to these can be found on our website. Note, we only cite the first mention of the resource within this paper.

## Methods

bioNerDS is designed and developed as an NER tool that aims to recognise database and software mentions in literature, and to provide a document-level “list” of resources mentioned in a given article. We identify resource names that represent databases, ontologies, classifications, software, programs, tools, web-services or packages, and exclude names of files and file formats, methods, algorithms, identifiers, operating systems and programming languages (see [9]).

**Figure 1:**
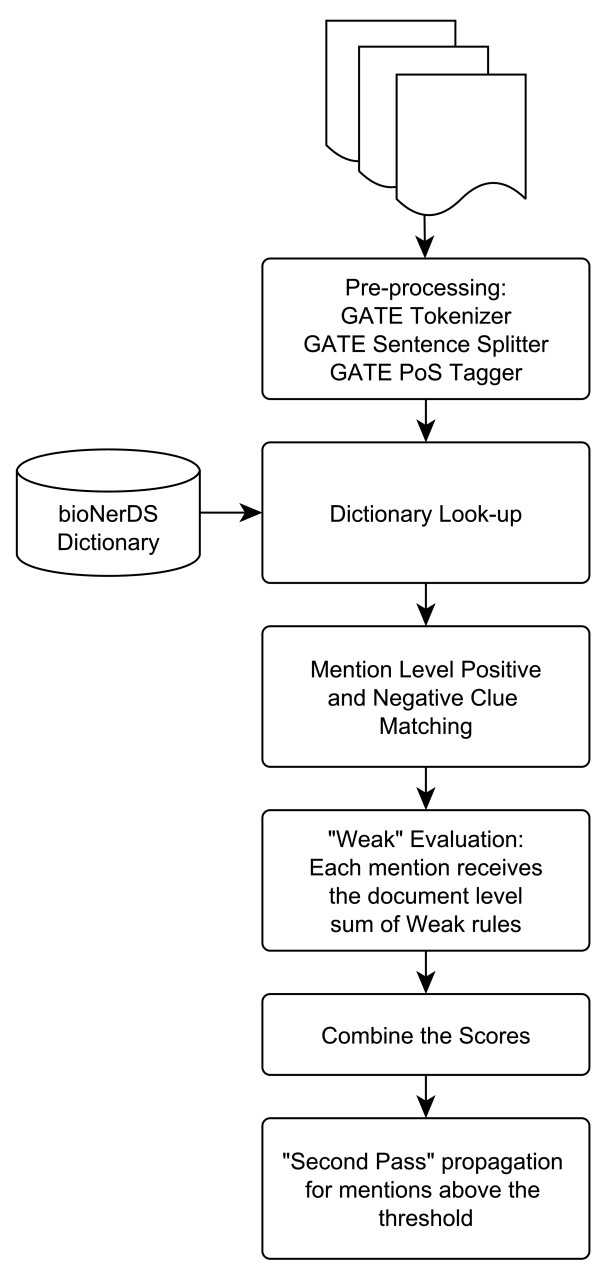
Flowchart of bioNerDS’ name recognition strategy.

Figure 1 represents a high-level overview of bioNerDS. Each document is first pre-processed using a typical text-mining pipeline consisting of tokenization, sentence splitting and part of speech tagging, all using GATE’s ANNIE plug-in [12,13]. In the following step, we apply a dictionary look-up to identify candidate mentions of tool/database names. Given the dynamic nature of the bioinformatics resourceome, the dictionary-based approach alone is insufficient for large-scale and dynamic capture of databases and software in the literature [9]. To increase coverage, we make use of several rule-based techniques to recognise unknown database and tool names in text. Therefore, in the third step, we use a number of local clues that indicate that a given phrase might represent a mention of a database/software name. Each mention is assigned a score; these scores can be positive or negative, depending on their indication of a true positive or a false positive match.

While previous steps focus on single mentions, we also collect supporting “weak” evidence across different mentions of the same candidate (within the document) and use it to update/adjust the mention-level scores. Finally, all candidate mentions with a score above a given threshold are propagated through to the whole document and extracted so a document-level list of mentioned resources can be generated. We discuss these steps in the following subsections.

### Dictionary lookup

At bioNerDS’ core is a large case-sensitive dictionary of known database and tool names. This dictionary contained 4,871 entries with 4,879 unique name variants compiled from several available online resource repositories and inventories (e.g., Nucleic Acids Research [14], the Bioinformatics Links Directory [15], Bioconductor [16] and Wikipedia [17]). Initial dictionary-matched candidates are assigned a score (see Table 1) that will be used to estimate our confidence that a given candidate is indeed a tool/database name. We note that names from Bioconductor have been assigned a small negative impact in addition to the initial dictionary score (see Table 1), as they are often homonymic with names of associated general bioinformatics concepts (e.g., *aCGH*[18], *affy*[19], *graph*[20], and *ROC*[21]). The dictionary was then used as input to LINNAEUS, a dictionary-based mention-level NER tool [22].

**Table 1:** Applied scores for local clues

**Pattern name**	**Description**	**Score**
Dictionary	Matches dictionary	+5.50
Title	Matches title pattern	+4.00
Enum	Is part of a known resource enumeration	+3.00
Hearst	Is part of a Hearst pattern	+4.00
“Good” Head	Associated with a positive head term	+2.00
Version	Followed by a version number	+3.00
Reference	Followed by a reference	+1.00
Hyper-Link	Followed by a hyper-link or URL	+1.50
Mixed Case	Is MiXeD CaSe	+1.00
Upper Case	Is UPPER CASE	+0.50
Bioconductor	Matches Bioconductor dictionary	-1.75
Dictionary Word	Is an English dictionary word	-4.00
Known Acronym	Is a known bio-acronym	-15.00
Negative Head	Associated with a negative head term	-15.00
Lower Case	Is lower case	-1.00
Partial-Word	Is only a part of a word	-15.00
Compound Factor	Term fires multiple positive clues	+0.50
Weak	Associated with a weak identifier	+0.50

bioNerDS’ various score adjustments.

### Applying local clues to recognise unknown mentions

We consider several situations where new/unknown database/tool names can be identified. In our previous research, we have shown that such names are typically contiguous sequences of nouns [9]. We find that including other part of speech tags in this definition is unhelpful, as names do not commonly include other token types (e.g., modifiers). Therefore, we focus on noun phrases that contain only nouns.

Each software or database candidate noun phrase mention is then assigned a score, which integrates scores for several clues that are spotted in the neighbourhood of the mention. Table 1 shows the individual scores assigned to particular patterns. Our approach is similar to other rule-based scoring approaches (e.g., species name identification [23]). Initial scores were generated by ranking the rules according to their extraction potential and assigning numeric values, with the most powerful given the highest score. We took these numbers as weights and adjusted them empirically based on results from the training data. The scores for all patterns that apply to a given mention are then summed up to provide a score for the given mention.

The positive local clues include the following: 

1. Title mentions: Many articles that introduce a new database or program place the name in their paper’s title, often following a standard format where the name begins the title, followed by some punctuation (colon, dash, etc.), and finally with a short description (or the expanded acronym) that typically includes a specific keyword. We have compiled a list of such keywords that indicate the presence of a software/database mention (e.g., *database, ontology, web service*, etc. [10,11]; a full-list is available from our website).

2. Enumerations and Hearst patterns: We have followed a standard approach to identify enumerations of names, primarily using Hearst patterns [24] — e.g., “*tool*such as*MUMmer* or *Vmatch*” (PMCID: PMC2753849), but also any list of noun phrases is considered, even if not part of a Hearst pattern, if at least one of the members has been recognised as a candidate name from our dictionary.

3. “Good” head nouns: The list of keywords is re-used in combination with the Stanford dependency parser [25] to identify noun phrases associated with the keyword heads (e.g., “*The PolyFreq*program”, PMCID: PMC1239908) in order to “recover” potential database and software names that precede the keyword.

4. Version mention: Strings that appear to represent a version number (e.g., *2.0*) following a candidate noun phrase are considered.

5. References and URLs: Such mentions are also good indicators of a possible preceding database or program name.

6. Positive orthographic clues: Words in *ALL CAPS* and *MiXeD CasE* gain a small positive boost in their score.

In case a mention matches several clues, they are all combined. Additionally, we take the number of different positive clues fired for the given mention, and multiply this by the Compound Factor, which is then added to the candidate’s score.

There are numerous resource names that are ambiguous; this is a common problem in NER tasks [22,26]. For example, the two most troublesome tool names that we came across were *Network*[27] and *analysis*[28], also widely used for common concepts in bioinformatics. We have therefore designed a set of rules that suggest a putative resource name would be incorrectly identified as a resource in a given context. The negative local clues include the following: 

1. Common English words and acronyms: Our primary method of filtering names is through term comparison to a common-English dictionary and an acronym list. If a predicted candidate term is either a known English word or known acronym, then it takes a score reduction (see Table 1). The English dictionary is derived from a publicly available list [29] and the acronym dictionary is derived from ADAM [30], consisting of 86,308 and 1,933 terms, respectively.

2. “Negative” head nouns: This approach is similar to the identification of keyword heads that characterise positive mentions, but instead uses a set of “blacklisted” terms with the primary aim of restricting the matches to only those within the scope of the definitions. For example, these heads help filter file formats, programming languages, methods, algorithms and so on (the full-list is available on our website).

3. Negative orthographic clues: *lower case* words receive a slight decrease in their final score.

4. A partial word match: This helps filter out some situations of incorrect tokenization, in particular, for database identifiers. For example, this will help filter out the “GO” in *GO:001234*.

All rules are designed in JAPE (compound regular expressions) and are matched using GATE [13].

### Cross-mention “weak” clues

In some cases, mentions of tools/databases do not have any of the “strong” clues mentioned above, but are rather used with specific verbs (e.g., *record*, *alignment*, *develop*, *ran*, *use*, *interface*, *platform*; see our website for the full list) or appear with some indicative, but ambiguous head (e.g., *interface*, *platform*). While these clues on their own are insufficient to suggest a resource name mention, when combined as weak clues across several mentions of the same candidate, they can be an indication of a resource. In this step we therefore calculate this combined score as the number of weak clues for that name throughout the entire document (all candidate terms within a document that map to the same putative resource, (e.g., all mentions of BLAST)). This value is multiplied by the score of the weak indicator (+0.50, Table 1) and then added to the individual score of each candidate term for that particular resource.

### Applying a threshold and document-level annotations

For each candidate mention whose total score is above a given threshold, all lexically equivalent mentions elsewhere in the document are also tagged. In the experiments reported in this paper, the minimum threshold a candidate term needed to exceed was +5.00. Such names are all added to an internal dictionary for that specific document, and LINNAEUS is then passed this “personalised” dictionary file to match against. In this way we “propagate” names that have been spotted with high confidence to mentions that do not have enough local clues on their own (mention-level propagation to document level). This is of particular importance in papers which have a heavy focus on one or two specific tools and these are then mentioned numerous times, often without other clues (e.g., not with a reference, or version number, or URL). As a result, the chance of bioNerDS matching all these mentions is small, leading to a substantially lower recall performance (see Results for validation).

## Results and discussion

### Evaluation of bioNerDS

A gold standard comprised of 60 full-text articles was split into a training set of 25 articles, a development set of 5 articles, an evaluation set of 25 articles (all from a random sample of *BMC Bioinformatics* and *PLoS Computational Biology* articles), and a set of 5 articles from *Genome Biology* that was used for evaluation only. A summary of the four corpora is provided in Table 2. The inter-annotator agreement (F-measure) for this corpus has been previously reported as 86% (80% strict; observed agreement 60%) [9].

**Table 2:** Corpora summary statistics

**Corpus**		**# of mentions**	
**Name**	**# of documents**	**Total**	**Unique**
Training	25	1074	189
Development	5	96	23
Genome Biology	5	245	40
Evaluation	25	1001	190

We make use of the standard evaluation metrics: precision (*P*), recall (*R*), and F-measure (*F*), based on the numbers of *true-positives* (*TP*), *false-positives* (*FP*), and *false-negatives* (*FN*):

(1) $$\begin{array}{ll} P=\frac{\text{TP}}{\text{TP}+\text{FP}} & \; \end{array}$$

(2) $$\begin{array}{ll} R=\frac{\text{TP}}{\text{TP}+\text{FN}} & \; \end{array}$$

(3) $$\begin{array}{ll} F=2\times \frac{P\times R}{P+R} & \; \end{array}$$

Table 3 shows the results with both strict (exact offset matches between gold standard and system annotation) and lenient matching (annotation overlap is sufficient; this is the main metric mentioned in the rest of the article). While we were able to obtain an F-measure of nearly 80% for the development set, the F-measure for the evaluation set was only 63%. For comparison, a baseline with dictionary-based approach achieved a lenient F-measure of just 54% [9]. After analysing the reasons behind the drop in performance, we concluded that there was a single document (PMC1664705) in the evaluation set responsible for a high number of total errors (32% of the total false positives): by excluding it, the lenient F-measure increased to 66%. The errors came from a series of false positive matches against “*P* and *D* modules”, where these protein modules were frequently used with *interface* and so were incorrectly matched by bioNerDS (protein docking interface, rather than a user/graphical interface). We also note that the performance on the Genome Biology evaluation corpus was more consistent with the training/development dataset, with an F-measure of 91%.

**Table 3:** Evaluation scores for bioNerDS

**Corpus**	**Mention level scores**			**Document level scores**		
	**Precision**	**Recall**	**F-Score**	**Precision**	**Recall**	**F-Score**
Training	0.82 (0.67)	0.73 (0.59)	0.77 (0.63)	0.75 (0.44)	0.70 (0.52)	0.72 (0.48)
Development	0.90 (0.69)	0.82 (0.64)	0.86 (0.66)	0.81 (0.46)	0.74 (0.55)	0.77 (0.51)
Genome Biology	0.93 (0.86)	0.89 (0.82)	0.91 (0.84)	0.82 (0.62)	0.74 (0.65)	0.78 (0.64)
Evaluation	0.58 (0.49)	0.68 (0.57)	0.63 (0.53)	0.65 (0.40)	0.60 (0.44)	0.63 (0.42)

*Lenient* agreement is provided for precision, recall and F-measure, with *strict* agreement in brackets.

Tools with ambiguous names are a source of lower precision, much like in other related NER tasks [22,26]. The context that disambiguates databases and software from other bioinformatics concepts proved hard to determine automatically. This is especially true of Bioconductor package names, which are often all in lower case, and use the same name as the corresponding approach, method or data that they are trying to provide (e.g., *aCGH*, *affy*, *graph*, and *ROC*). This problem also includes other resources such as the database *tRNA*[31] listed in the BMC Databases catalog [32], and the ambiguously named *analysis* and *Network* programs. Although bioNerDS contains rules to help address these (through a score reduction), they can still be scored high enough to pass the threshold based on other presented evidence.

Fuzzy distinctions between tools, methods and algorithms, and of file formats and programming languages alongside naming inconsistencies between authors also caused some false positive and false negative results in our evaluation.

### Effects of cross-mention scoring

To evaluate the effectiveness of the weak clues and score propagation approach, bioNerDS was run on the three corpora (training, evaluation and Genome Biology) with rules for various clues disabled. This allowed us to determine how much of the final accuracy is attributed to each of the clues. We note, however, that no module scores or thresholds were retrained or adjusted for these tests. Table 4 shows the mention-level results for three different settings. They are all having a positive impact to the overall F-score from the corpora. In the cases of weak clues and score propagation, this is down to the large effect that they have on the recall of the system, providing increases ranging from 5 to 33%. As can be expected, they do generally provide some negative impact on precision (up to 12%), but the overall impact on F-score was a positive one in each case. Conversely, disabling the “head pattern” gave mixed results. For the training set, disabling it increased both recall and precision, resulting in a 3% higher F-score. In the evaluation and Genome Biology corpora, disabling it resulted in reduced F-scores (by 2% and 6%).

**Table 4:** bioNerDS evaluation scores with some clues disabled

		**Without head**	**Without weak**	**Without score propagation**
	Recall	0.78	0.63	0.68
Training	Precision	0.83	0.83	0.85
	F-score	0.80	0.72	0.76
	Recall	0.65	0.50	0.53
Evaluation	Precision	0.57	0.70	0.54
	F-score	0.61	0.58	0.54
	Recall	0.88	0.73	0.56
Genome Biology	Precision	0.82	0.92	0.89
	F-score	0.85	0.82	0.69

Standard lenient mention level evaluation scores for bioNerDS on different corpora with various detection modules disabled.

### Literature analysis

Few literature surveys of bioinformatics resource usage currently exist. One such example by Southan and Cameron [33] surveyed the mentions of databases in the literature, but focused only on European nations and articles from the previous 10 years with “database” in their titles. For our survey of software and database usage, we applied bioNerDS to the entire collection of BMC Bioinformatics and Genome Biology full-text journal articles (up to 2011) as downloaded from PubMed Central (PMC) [34]. We selected these two open-access journals as BMC Bioinformatics aims to provide a venue for publishing about resources for bioinformatics research, whereas Genome Biology’s remit is to apply bioinformatics tools to gain biological insight, which seemed a good contrast for comparison. Each journal’s associated scope emphasises this assessment [35,36]. The experiment was performed on a total of 6,267 full-text open access documents, with 3,746 from BMC Bioinformatics and 2,521 from Genome Biology. A small subset of these were excluded (84 from BMC Bioinformatics and 55 from Genome Biology) due to full-text files that were unavailable at the time or because preprocessing text mining tools were unable to process them.

Before this experiment, we updated the primary dictionary used in bioNerDS by both including all the terms annotated in the gold standard sets, and by updating all of the dictionary file lists to 28th February, 2012 from 12th April, 2011. This resulted in roughly 1,400 additional entries being added to the dictionary (if this updated dictionary is applied to the evaluation set, an F-measure of 72.5% is achieved; 88% recall).

In total, Genome Biology contained over 53,000 mentions (5,284 unique), and BMC Bioinformatics contained over 174,000 mentions (with 13,023 unique). The results revealed that 77.6% of the Genome Biology papers contained at least one resource mention, compared to 97.7% of BMC Bioinformatics papers. Table 5 provides the mean number of documents to contain a mention for the top 10 resources of each journal. Both journals include many of the well-known and well-established databases and tools within the bioinformatics field (e.g., R [37], Gene Ontology [38], BLAST and GenBank [39]). However, some interesting differences between the two journals emerged, with Genome Biology featuring Ensembl [40], whereas BMC Bioinformatics featured PDB [41], KEGG [42] and MySQL [43] within the top 10. There are also some probable errors resulting from bioNerDS’ possible false-positive hits (e.g., *tRNA*, *S4*[44], *Q*[45] and *Network*).

**Table 5:** Top 10 resources and the mean number of documents to include them

**Genome biology**		**BMC bioinformatics**	
R	0.18	R	0.36
Gene Ontology	0.17	Gene Ontology	0.19
BLAST	0.15	BLAST	0.17
GenBank	0.14	PDB	0.09
GEO	0.09	GenBank	0.09
Ensembl	0.08	Q	0.08
tRNA	0.07	Network	0.08
S4	0.07	MySQL	0.11
Cluster	0.06	KEGG	0.08
RefSeq	0.05	GEO	0.08

The full document level lists are available on our website.

Table 6 provides the results obtained on the mention level for the top 10 resources from each journal. It features many of the same resource names listed as in Table 5, but some notable changes are that KEGG now appears in both journals’ top 10 lists, and SCOP [46] and PubMed [47] now appear in BMC Bioinformatics. Additionally, Gene Ontology has overtaken R in both journals. Both tables also show that BMC Bioinformatics tends to have higher overall counts of resource mentions than Genome Biology, with the notable exceptions of GenBank, Ensembl and GEO [48], which are all higher in Genome Biology.

**Table 6:** Mean number of mentions within a document of a top 10 resource

**Genome biology**		**BMC bioinformatics**	
Gene Ontology	1.45	Gene Ontology	2.80
R	0.58	R	1.46
BLAST	0.49	BLAST	0.87
GenBank	0.42	PDB	0.74
Ensembl	0.34	SCOP	0.50
GEO	0.24	Q	0.41
RefSeq	0.21	KEGG	0.39
HGT	0.21	Pfam	0.33
KEGG	0.20	PubMed	0.29
miRNA	0.19	GenBank	0.28

The full mention level lists are available on our website.

Mention level counts are, as expected, higher than document level counts, and this is likely to be due to one of a few reasons. First, a resource could be mentioned in the, Background, Methods and Results and discussion sections giving an average of 4-5 times per document. Alternatively, the same database could be used to annotate multiple data entries within a single document, or used for more than one entry/record from that database (e.g., with GO, PubMed, GenBank and Ensembl). R also has very high mention level counts — this could be because it can be used for multiple different analyses (in combination with Bioconductor, for example) and because it is picked up as both a resource, and as a programming language (which is a false-positive by our definition). Conversely, a resource might have relatively low mention level counts compared to document level counts if it is a programming resource, and thus only relevant to a particular part of the articles underlying method (e.g., MySQL). Finally, multiple mentions of a single resource within a paper can be due to a comparison between two (or more) resources made within that paper (for example, comparing BLAST, ClustalW and MUSCLE [49] multiple-alignment tools).

**Figure 2:**
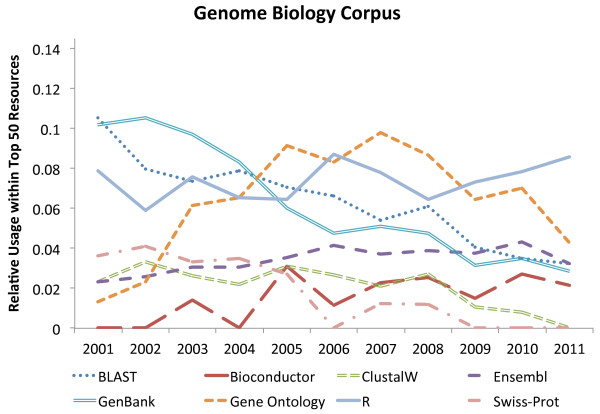
**Relative usage of top resources in Genome Biology over time.** Highlights the relative usage of some well known bioinformatics resources within the top 50 resources used at document level within Genome Biology.

**Figure 3:**
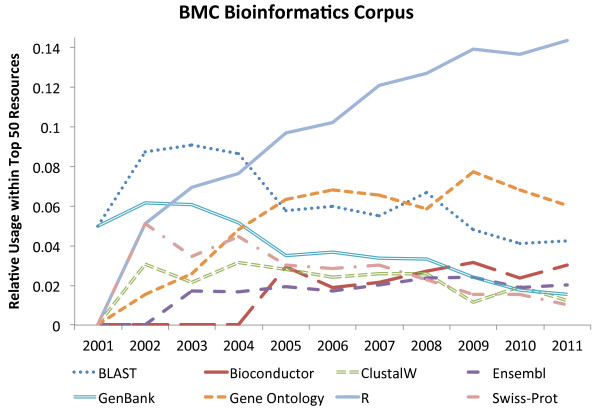
**Relative usage of top resources in BMC Bioinformatics over time.** Highlights the relative usage of some well known bioinformatics resources within the top 50 resources used at document level within BMC Bioinformatics.

To evaluate how resource usage has changed over time, we selected some key database and software resources and plotted the relative use (document level mentions in a given year, divided by the total number of journal papers in that year,) for each year from 2001 to 2011 inclusive for each journal on the document level. The resulting graphs (Figures 2 and 3) show that R usage (and, perhaps by relation, Bioconductor) has been consistently increasing for both journals over the 10 year period (more so within BMC Bioinformatics) and that the use of Gene Ontology has steadied off in recent years after a rapid increase in the first few years (of the survey). Most other major resources have started to decline in usage for both journals (e.g., GenBank, Swiss-Prot [50], BLAST and ClustalW) and this decline is more prevalent in BMC Bioinformatics than in Genome Biology. Finally, in contrast to other database resources, Ensembl has seen a slight increase in usage in Genome Biology.

The large overall usage of R and Gene Ontology within both journals is of interest. This suggests that R is now being accepted as the standard statistical analysis resource for biology and bioinformatics, and that the Gene Ontology is quickly becoming the primary shared vocabulary in the field and is an important resource for bioinformatics and computational biology. The observed relative decline in usage of some “top” resources can, perhaps, be explained by the continued increase in the number of resources becoming available over time, and — as new ones are developed — older ones are slowly phased out. An example is the increase in the use of MUSCLE as an alternative to ClustalW. Similarly, Swiss-Prot fails to feature in the top 50 list for Genome Biology post 2008, possibly as people started to cite Uniprot (a combination of Swiss-Prot and TrEMBL) [51].

Other interesting findings include, for example, that, for both journals, MUSCLE has only appeared in the top 50 in recent years with a steady increase in ranking. GEO has seen a rise in usage in Genome Biology, while BMC Bioinformatics has MeSH, PubMed and MEDLINE featured much higher than in Genome Biology, suggesting a greater focus on more general biological computational methods and analysis, and a wider bioinformatics scope that, for example, includes text-mining and semantic indexing. Conversely, Genome Biology features Galaxy [52] within its top 50 (mention level), whereas BMC Bioinformatics does not (ranked 1369). This suggests that Genome Biology articles are more focused on data analysis using established techniques rather than on introducing novel ones.

**Figure 4:**
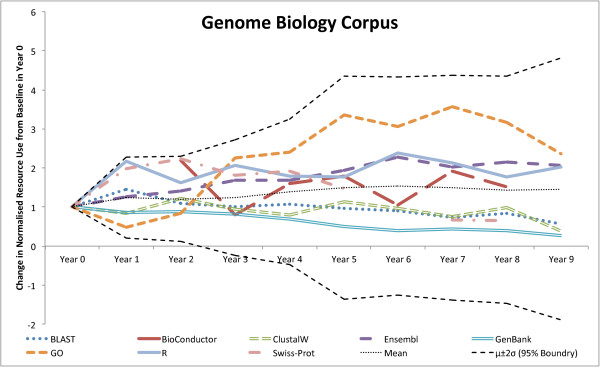
**Genome Biology’s upper and lower 95% bounds.** Comparison of a resource’s change in relative use, compared to the expected change based on a random walk using a Gaussian distribution fitted to the normalised resource usage changes from a baseline in Year 0 for Genome Biology. The upper and lower 95% bounds are calculated as two standard deviations from the mean.

**Figure 5:**
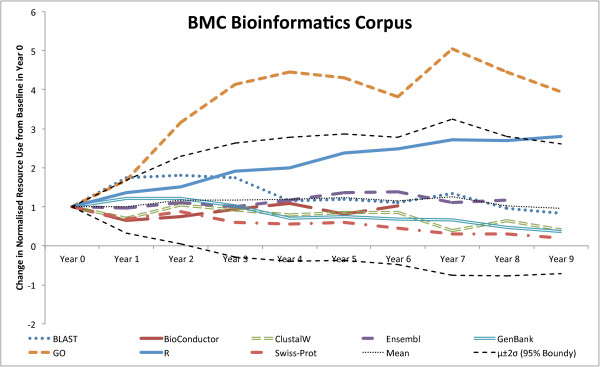
**BMC Bioinformatics’s upper and lower 95% bounds.** Comparison of a resource’s change in relative use, compared to the expected change based on a random walk using a Gaussian distribution fitted to the normalised resource usage changes from a baseline in Year 0 for BMC Bioinformatics. The upper and lower 95% bounds are calculated as two standard deviations from the mean.

To test whether the variations seen in usage were those that we might expect by chance, we looked at the normalised usage statistics of the resources and compared the change in usage starting from a normalised baseline (Year 0). Across the resources, the changes in usage had an approximately Gaussian distribution. We could therefore test whether the usage patterns seen across years mapped onto what would be expected from a Gaussian random walk process [53]. The expected variation (95% confidence interval) of the random walk process was therefore added to the graph of the normalised differences. If the variation graphs stayed within these bounds we could infer that the changes could be accounted for by a random walk process. Deviations from these bounds would suggest a process different from a random walk. The resulting graphs showing the results of these analyses are given in Figures 4 and 5. For Genome Biology, there is no significant deviation from the 95% boundaries, suggesting that the variation could be down to random variation alone. However, within BMC Bioinformatics, the Gene Ontology is increasing at a rate that far surpasses the upper 95% boundary, confirming that the Gene Ontology has seen a significant rise in usage over the last 10 years (at least, within BMC Bioinformatics). R is also trailing close to the upper boundary, also suggesting that an external factor outside of general variation is having an influence on its usage. We note that the Gene Ontology is increasing at a higher rate than R in Figure 5, whereas in the initial graph (Figure 3), R is growing faster than the Gene Ontology. This is the case because the Gene Ontology has a lower starting value and so, when normalised by Year 0, sees a higher relative rate of growth. The data used for these tests is provided in Additional file 1.

To further explore the “rate” of change in the bioinformatics resourceome in the two journal corpora, we plotted the normalised frequencies of each resource per year against the sum of the absolute difference (*ΣΔ*) in those frequencies across the years. So, for the number of mentions *x*^*y*^ of a resource *x* in a given year *y*, we define:

(4) $$\Sigma \Delta =\overset{2010}{\underset{y=2000}{\sum }}|x^{y+1}-x^{y}|$$

**Figure 6:**
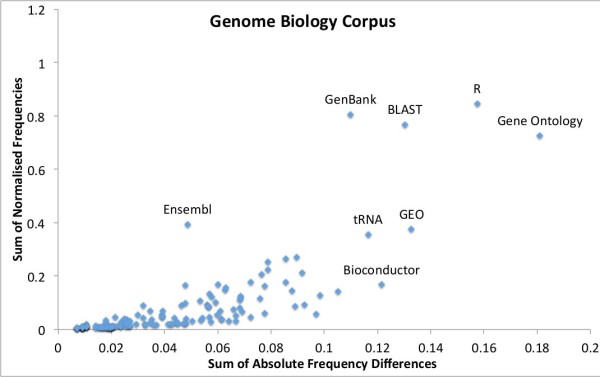
**Genome Biology’s variation in top 50 resource usage.** The sum of normalised frequencies against the sum of absolute differences for Genome Biology’s top 50 resource mentions with interesting outliers labelled. The *y* axis highlights the relative level of use of a resource, whereas the *x* axis shows the level of variation of tool use across the years 2000 to 2011.

**Figure 7:**
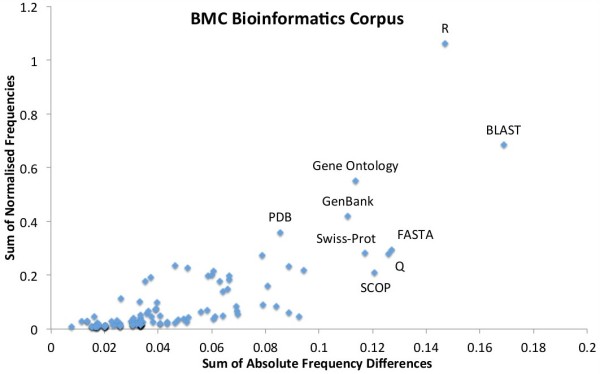
**BMC Bioinformatics’s variation in top 50 resource usage.** The sum of normalised frequencies against the sum of absolute differences for BMC Bioinformatics’s top 50 resource mentions with interesting outliers labelled. The *y* axis highlights the relative level of use of a resource, whereas the *x* axis shows the level of variation of tool use across the years 2000 to 2011.

The graphs in Figures 6 and 7 show that, when comparing the two journals, Genome Biology has higher usage counts of Gene Ontology and GenBank, whereas BMC Bioinformatics has higher usage of R. This seems to be in contradiction to the results in Tables 5 and 6, but we note that the tables are normalised for the whole corpus (per document), whereas the graphs are normalised by the total resource counts of the top 50 resources. Conversely, Genome Biology has a higher variation in use (higher *ΣΔ*) of Gene Ontology and R, with BMC Bioinformatics having a higher variation in BLAST use. It is also of interest that Bioconductor and GEO feature to the right of the Genome Biology graph confirming their rapid acceleration of usage in recent years (high variation).

With Genome Biology’s greater emphasis on biological insight and biological data over method development, it is no surprise that it gets higher absolute usage results for GenBank (both per journal and per article), and higher normalised counts for GenBank, BLAST and Gene Ontology. This helps suggest a potential common methodological pattern of “computational biology”: get sequence (GenBank and Gene Ontology), characterise and analyse (Gene Ontology) and compare it (BLAST). On the other hand, BMC Bioinformatics “favours” R usage, which could be down to an inherent use of R in Genome Biology (to do statistics, generate ROC curves, etc.), compared to the general use of R as a programming platform in BMC Bioinformatics for method development. Genome Biology also has a wider usage of Gene Ontology (high *ΣΔ*). This could be because Genome Biology can (as with R) apply the result of a Gene Ontology/R analysis once wrapped up as another tool or database without needing the direct reference, e.g., during an over-expression analysis. Finally, BMC Bioinformatics has high variation in the use of BLAST, which is often used as a comparison for new tools, whereas it would tend to form part of a primary analysis pipeline within Genome Biology articles.

The data also show that 25.3% of BMC Bioinformatics papers potentially mention a new resource in the title, as opposed to only 4.3% of Genome Biology papers, confirming again that BMC Bioinformatics has a much greater focus on resource creation than Genome Biology.

We additionally calculated the resource name union and intersection between the two journals. The intersection covers 34% of the resource mentions in the Genome Biology corpus and 14% of the BMC Bioinformatics corpus. Only 11% of all resource names collected are contained within both journals. These names, however, accounted for 57% of the total mentions extracted. This further highlights how a relatively small number of resources are mentioned very frequently within (and across) the literature. Conversely, 53% of the total number of unique resource names extracted across both journals were only mentioned once (at the mention level).

Finally, we evaluated the “long tail curve” property of our data given the hypothesis that a majority of resources are introduced, but hardly used again and potentially ignored after that point. We are careful not to extrapolate too much from this analysis as our results are only from two journals. The document level results reflect this hypothesis (see Additional file 2): for example, 95% of resources are mentioned less than 6 times (78% only once) and, on the other end, the top 100 resources account for over 96% of all mentions (9% of the total mentions are of R). There was little difference between the two journals for these figures.

We note several limitations of the analysis presented here. Within this survey, we have considered a resource mention to imply the use of that resource, though we are aware that this is not always the case. There are also several other limitations, due to the nature of the topic. Firstly, we do limited resource aggregation of name variants for our survey — in particular, we aggregate some known name variants involving word-case and acronyms (as automatically recognised by LINNAEUS or BADREX [54]), those linked in our primary dictionary, and the use of spaces verses dashes/hyphens (it is perhaps important to point out that this aggregation combines matches for ClustalW with matches for ClustalX in text, which we have only referred to as ClustalW within this paper). For a fairer analysis, more extensive name normalisation would be required on the data to accurately group all name variations. Second, our study does not directly take into account the creation date of resources. We would expect that resources that have been around longer, would generally have more mentions. However, as our analysis only goes back as far as the journals we are looking at, normalising by the number of years that we have mentions for would be unfair. Finally, although we normalise for the number of articles processed in a given year, this does not take into account the number of alternative places to publish in that year, or the general increase in publication rate each year. A far more detailed analysis of the types of trends and their potential reasons, particularly using the resources to characterise the journals is needed, similar to the review of biomedical corpora usage by Cohen *et al.*[55].

## Conclusions

bioNerDS can recognise mentions of bioinformatics’ databases and software in primary literature with a reasonable accuracy. It achieved an F-measure of between 63% and 91% on different datasets (63%–78% at the document level). Though other NER tasks, like gene name recognition, are now considered mature, this was not always the case, especially when gene recognition was first attempted. For example, in the first BioCreAtIvE task, F-measures ranging from below 50% to just over 80% at best were achieved [26]. bioNerDS is, to the best of our knowledge, the first attempt at comprehensive database and software name recognition at the mention level and identification accuracy will improve. While further work is required, we think that the approach represents a significant step towards providing a means to explore the usage of databases and tools in bioinformatics.

Still, the accuracy achieved is sufficient to evaluate resource usage across the literature on both the document and mention levels. We have further demonstrated the potential of bioNerDS in exploring similarities and differences between journals and fields through systematic literature analysis of database and software use. The results obtained provide an indication of the similarities and differences between the two journals surveyed.

Finally, additional work is required both to further increase the accuracy of the tool (especially in automated recognition of false-positive results) and in a more comprehensive analysis of the results obtained.

bioNerDS and the data extracted are available at http://bionerds.sourceforge.net/ under the Simplified 2-Clause BSD Licence.

## Abbreviations

ADAM: another database of abbreviations in MEDLINE; ANNIE: a Nearly-New Information Extraction System; bioNerDS: bioinformatics Named entity recogniser for Databases and Software; BIRI: BioInformatics Resource Inventory; BLAST: Basic Local Alignment Search Tool; BMC: BioMed Central; F: F-score/F-measure; FN: False Negatives; FP: False Positives; GATE: General Architecture for Text Engineering; GEO: Gene Expression Omnibus; GO: Gene Ontology; HGT: Horizontal Gene Transfer (name of a database or name of a program, or could just be a false positive hit); JAPE: Java Annotation Patterns Engine; KEGG: Kyoto Encyclopedia of Genes and Genomes; MeSH: Medical Subject Headings; NER: Named entity recognition; OReFiL: Online Resource Finder in Life sciences; P: Precision; PDB: Protein Data Bank; PMC: PubMed Central; PMCID: PubMed Central Identifier; R: Recall (also used for the statistics program R, which is not an acronym); S4: Structure-based Sequence alignments of SCOP Superfamilies; SCOP: Structural Classification of Proteins; TP: True Positives.

## Competing interests

The authors declare that they have no competing interests.

## Authors’ contributions

GD programmed and ran bioNerDS on the Genome Biology and BMC Bioinformatics journal corpora, and drafted the manuscript. AB helped with the data analysis. GN, DLR and RS initially conceptualised the project and provided continual guidance and discussion. All authors read and approved the final manuscript.

## Supplementary Material

Additional file 1 **Genome Biology and BMC Bioinformatics temporal analysis statistical evaluation data** Excel spreadsheet containing the normalised top 50 resource mentions for each journal, and the resulting mean and standard deviations for each year, used to generate Figures 4 and 5. It also contains the plotted data curves showing the approximate Gaussian distribution of the data. Click here for file

Additional file 2 **Genome Biology and BMC Bioinformatics dataset** Excel spreadsheet containing the top 50 resources mentions for each journal by year on the document level as well as the resulting graphs used within this paper. Also includes long tail curve analysis data. Click here for file
